# An assessment of the breastfeeding practices in Momo division, North West region of Cameroon

**DOI:** 10.1002/fsn3.1808

**Published:** 2020-08-10

**Authors:** Boh Nwachan Mirabelle, Aba Ejoh Richard

**Affiliations:** ^1^ Department of Nutrition, Food and Bioresource Technology College of Technology University of Bamenda Bambili Cameroon

**Keywords:** breastfeeding information, breastfeeding practices, Cameroon, Momo division

## Abstract

The aim of this study was to assess maternal breastfeeding practices of children aged 0 to 24 months in Momo Division. It was also to identify the maternal sources of breastfeeding information and determine their impact on maternal breastfeeding decision in this division. Structured interviewer questionnaires were administered to 540 mothers. Among the 540 children included in the study, 77% of them received no prelacteal foods. Despite the fact that 47% of mothers initiated breastfeeding early, only 38% exclusively breastfed their infants for 6 months. Complementary feeding was introduced before the age of 6 months (59.5%). Few infants (2.4%) received complementary feeding later than 6 months. Only 3.1% of the children were breastfed up to two years or beyond. The most common source of breastfeeding information for the mothers is from the family, friends, and neighbors but information from healthcare professionals had the most influential effect on maternal breastfeeding decision. Although a few mothers in Momo Division followed the recommended breastfeeding practices, the breastfeeding practices in this division are generally suboptimal.

## Graphical abstract

The aim of this study was to assess the maternal breastfeeding practices of children aged 0–24 months, to identify the maternal sources of breastfeeding information, and to determine their impact on maternal breastfeeding decision in Momo Division. Although a few mothers in Momo Division followed the recommended breastfeeding practices, the breastfeeding practices in this division are generally suboptimal. The most common source of breastfeeding information for the mothers is from the family, friends, and neighbors, but information from healthcare professionals had the most influential effect on the maternal breastfeeding decision.

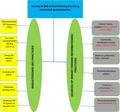

## 1 INTRODUCTION

Malnutrition is a public health problem worldwide associated with a heavy burden of infant morbidity and mortality. Child malnutrition is most prevalent in West and Central African countries including Cameroon (UNICEF, WHO & World Bank Group, 2020). Childhood malnutrition is a public health problem in Cameroon, particularly among infants whose age ranges between 0–59 months and the death of 45% of children in this age group is linked to malnutrition (UNICEF Cameroon, 2015). During the first two years of life, poor feeding practices are among the major causes of malnutrition (Bakalemwa, 2014). Proper evidence‐based feeding practices are indispensable for attaining and maintaining a good nutritional status, growth, development, and health and thus the very survival of children.

Breastfeeding (BF) plays a substantial role in improving nutrition, education, and maternal and child health and survival. Optimal BF practices during the early years of life have been recognized for many decades as one of the most cost‐effective interventions in reducing infant and child morbidity and mortality globally (WHO & UNICEF, 2017). Breastfeeding is not only the most cost‐effective method of nutrition, but is also a unique way of providing ideal nutrition as breast milk contains all the nutrients needed by the infant for healthy growth and development. BF is of great significance for the infant as it has been associated with increased intelligence, cognitive ability, school performance, productivity, earning ability, and social development. Thus, BF has the single largest potential impact on child morbidity and mortality of any preventive intervention. It also provides infants with superior nutritional content that is capable of improving the immunity and possible reduction in future healthcare spending (WHO, 2017). Breastfeeding is outstandingly imperative for developing countries, where child malnutrition is rising and childhood diseases such as diarrhea, pneumonia, and measles are very rampant. EBF bestows immunity against these illnesses and decreases children's risk of becoming overweight or obese (WHO, 2017). Nearly half of all diarrhea episodes and one‐third of all respiratory infections would be prevented with EBF (Victora, Aluisio, Barros, & Franca, 2016).

The WHO and UNICEF (2017) recommend that women should initiate BF within 1 hour after birth and exclusively breastfeed their infants for the first 6 months of life. BF should continue into the second year of life or longer with appropriate and sufficient complementary food. Although the WHO's recommendation on BF practices has been in effect for over three decades (WHO/UNICEF, 1989), most women do not comply with it as in developing countries, only 39% of infants benefit from BF up to 2 years of age and only 38% of infant age 0–5 months are exclusively breastfed (Cai, Wardlaw, & Brown, 2012). This low adoption of appropriate BF practices has been persistent, especially in Africa and Cameroon there is no exception. The rate of exclusive breastfeeding (EBF) in West and Central Africa (28%) remains among the lowest in the world (Bora, 2016).

Although the prevalence of BF in Cameroon is high (97%), suboptimal BF practices still prevail as the percentage of infants who receive timely initiation of BF (within 1 hr after delivery) is only 30% and 20% are exclusively breastfed (Demographic and Health Survey and Multiple Indicators Cluster Survey DHS‐MICS 2011). The rates of EBF in Cameroon keep on declining as the years go by. In 2004, it was 24%, while in 2011, it dropped to 20% and continued to 18% in 2016 (Istitut National de la statistique‐INS/Cameroun and ORC Macro, 2005; Demographic and Health Survey and Multiple Indicators Cluster Survey DHS‐MICS, 2011; Fombong et al., 2016).

Existing evidence has revealed that BF practices vary in different regions and communities of the country. In Bangang rural community, Cameroon, 69% of children are breastfed with only 23% of them exclusively breastfed (Mananga, Kana‐Sop, Nolla, Tetanye‐Ekoe, 2014) and in the Okola Health District, Cameroon, the EBF rate is 10.5% (Ngameni, Nana, & Adie, 2011). In the West Region, the prevalence of BF is 99%. Early initiation of BF and EBF was practiced by 7.8% and 20% of the mothers, respectively (Chiabi et al., 2011). In the North West Region (NWR), Lem (2015) compared BF practices between a rural area (Bafut) and an urban area (Bamenda), but information on BF practices in Momo division has not been documented.

Of all BF interventions to promote, protect, and support BF, information and education are the most effective (WHO & UNICEF, 2003). Some of the sources of information used as interventions to promote optimum BF practices by the government of Cameroon include the following: BF education and training programs through institutions such as health facilities and schools, the media, and community mobilization events and a legislation to protect the BF rights of working women (World breastfeeding Trends Initiative Cameroon, 2013). The source of information and its accuracy is increasingly recognized as vital tools in the BF decision‐making process of any mother, influencing it differently (Andrew & Harvey, 2011; Imdad, Yakoob, & Bhutta, 2011; Kornides & Kitsantas, 2013). It is therefore important to assess the impact of these strategies, as well as other sources of information on the maternal BF decision in this division. This study was to assess the maternal BF practices of children aged 0–24 months as well as identify the maternal sources of BF information and determine their impact on maternal BF decision in Momo Division, NWR of Cameroon.

## 2 MATERIALS AND METHODS

### 2.1 Study area

The study was conducted in 22 health facilities in Momo Division. Momo Division, one of the seven divisions in the NWR of Cameroon, is inhabited by 138, 693 people, with a population density of 77.40 inhabitants per km^2^. The majority of the population are farmers, semi‐skilled, or unskilled laborers. This division is divided into five subdivisions: Batibo, Mbengwi, Ngie, Njikwa, and Widikum with its head quarter being Mbengwi (North West Regional Agency of the National Institute of Statistics in Bamenda, 2017). It has a surface area of 1792 km^2^. The road network in this division is very poor, and because of this, the status of health facility is also poor (North West Regional Agency of the National Institute of Statistics in Bamenda, 2017).

### 2.2 Research design, recruitment of participants and data collection

The study utilized a descriptive cross‐sectional study design to assess the BF practices in this division. The survey was carried out from August to November 2017.

A sample of 540 mothers/child couples were randomly selected from all the five subdivisions of Momo. The children were aged between 0 and 24 months and were either BF or not at the time of the study. The number of mothers included in the study exceeds that expected from Fishers formula for sample size (Fisher, Laing, Stoeckel, & Townsend, 1991).

The study participants were mothers who came to the health facility either for pediatric consultations or for vaccination of their children and gave their informed consent to participate in the study. A pretested structured interviewer questionnaire was used to collect data from the study participants.

The questionnaire consisted of questions on socio‐demographic information and the actual practices of BF such as foods given before the first breastfeed, time of BF initiation and time of introducing complementary feeding and duration of BF. Also assessed were reported reasons for being able to practice EBF or not, such as the need to return to work or school, or baby not being satisfied by breast milk only. Recommendations regarding optimal BF practices by the WHO were used as a gold standard to compare the mothers' BF practices. The questionnaire also comprised of questions requesting the mothers to identify their sources of BF information and the sources that influenced their BF decision the most.

### 2.3 Data processing and analysis

After collecting the data, the database was then cleaned and a code was ascribed to each data. The data were entered using Microsoft Excel 2011. The data were transported to SPSS version 20.0 for statistical analysis. Frequency distributions, bar charts, and tables were produced using Microsoft Excel 2011.

## 3 RESULTS AND DISCUSSION

### 3.1 Demographic characteristics of the mother and baby

The demographic characteristics of the mothers and their babies are shown in Table 1. Most of the mothers (74%) were aged below 30 years. Educationally, most of the respondents attended primary and secondary schools with 38.9% and 46.1% respectively. Table 1 showed that most of the women give birth in health units (88.7%), and most of them are self‐employed (62.2%). About three‐quarters of the women (75.4%) were married, and there were slightly more females (51.5%) than males (48.5%). Most of the children were aged between 0–6 months (61.5%).

**Table 1:** Demographic characteristics of the mother and baby

Characteristics	Category	Frequency	Percentage (%)
Age of the mother	Below 30 years	400	74.1
	31–40 years	126	23.3
	Above 40 years	14	2.6
Highest level of education	No formal education	14	2.6
	Secondary education	249	46.1
	Primary education	210	38.9
	Higher education	67	12.4
Place of delivery	Health unit	479	88.7
	At home	61	11.3
Employment status	Paid job	52	9.6
	Self‐employed	336	62.2
	Unemployed	152	28.2
Marital status	Married	408	75.4
	Never married	113	20.9
	divorce	13	2.4
	Widow	6	1.3
Marital status	Married	408	75.4
	Never married	113	20.9
	Divorce	13	2.4
	Widow	6	1.3
Sex of the baby	Male	262	48.5
	Female	278	51.5
Baby's age (in months)	0–6	332	61.5
	7–13	151	28
	14–24	57	10.6

### 3.2 Breastfeeding practices

#### 3.2.1 Prelacteal feeding

About 77% of mothers did not give any prelacteal foods to their children but fed the children with colostrum. Over 23% of the mothers gave prelacteal food to their babies within the first 3 days after delivery, thus not respecting the recommendations of WHO which indicates that prelacteal foods should not be given to infants (WHO, 2017). This mal practice delays BF initiation and thus prevents the baby from enjoying the benefits of early initiation which include immunity against infections (El‐Gilany, Sarraf, & Al‐Wehady, 2012). Chiabi et al. (2014) found that 14.2% of the mothers at the Yaoundé Gynaeco‐Obstetric and Pediatric Hospital, Cameroon gave prelacteal foods to their children while a higher rate (30.36%) of prelacteal feeding was found in the West Region of Cameroon (Chiabi et al., 2011). The results of this study are in line with an earlier finding in the southwest Region of Bangladesh which revealed that 21% of studied mothers gave prelacteal foods to their babies (Islam, Rahman, Kamruzzaman, Islam, & Samad, 2013). In Momo Division, those who gave prelacteal foods to their babies gave precisely water (7%), infant formula (5%), and glucose (11%). These same types of prelacteal foods were given to by women who delivered at the Yaounde Gynaeco‐Obstetric and Pediatric Hospital Cameroon (Chiabi et al., 2014).

##### Reasons for introducing prelacteal feeding

**FIGURE 1:**
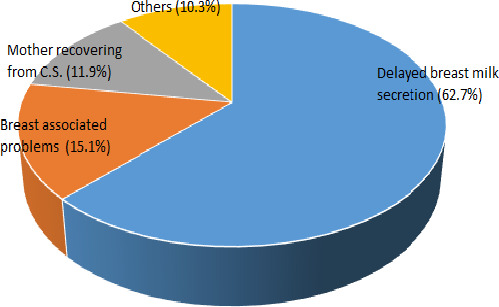
Reasons for introducing prelacteal feeding

The reason for introducing prelacteal foods (Figure 1) were delay in breast milk secretion (62.7%), problems associated with the breast (15.1%), mother recovering from cesarean section (11.9%), and other factors were the reasons of introducing such food to the infant (10.3%).

Similar reasons were indicated for prelacteal feeding in southwest Nigeria (Akinyinka, Olatona, & Oluwole, 2016). However, in rural Northern Ghana, Bafut subdivision of the NWR of Cameroon and Emirati, the reasons for prelacteal feeding were common and included hunger, colic, traditional beliefs, and hydration (Aborigo et al., 2012; Lem, 2015; Radwan & Roger, 2016, respectively).

#### 3.2.2 Breastfeeding initiation

The initiation of BF was suboptimal. Out of the 540 participants, 252 (47%) initiated breastfeeding within the first hour after parturition as recommended by WHO and UNICEF (2017), 37% initiated between 1–23 hr, and the remaining 15% initiated BF after 23 hr implying 53% of the mothers failed to adhere strictly to the WHO recommendation. This rate of early initiation of BF within 1 hour of birth is slightly higher than the 40% reported by the Global Nutrition Report (2018) for Cameroon. These findings are in line with the report by Dash & Choudhury (2005), where 48% of babies were breastfed for the first time within 6 hours following birth. Higher rates of timely initiation have been recorded in areas like Barrack in southwest Nigeria (56.5%), Bangladesh (61.1%), Da Nang, Viet Nam (78.7%), Turkey (62%) (Akinyinka et al., 2016; Islam et al., 2013; Nguyen et al., 2018; Yesildal et al., 2008, respectively). This may be as a result of better and more efficient BF information in these regions.

#### 3.2.3 Time of introduction of complementary foods

Inappropriate introduction of complementary food before the age of 6 months was a common practice in this division (Table 2). It was observed that 59.5% of the mothers introduced complementary foods before the infant was 6 months of age. Only 38.1% of children started complementary feeding at 6 months. A very small proportion of the infants (2.4%) received complementary feeding later than recommended as they received it when they were older than 6 months.

**Table 2:** The breastfeeding practices of the mothers

Bf practices	Category	Frequency	Percentage (%)
Prelacteal feeding	Water	41	7.6
	Infant formula	24	4.4
	Glucose	61	11.3
	Total	126	23.3
Time of BF initiation	Less than an hour	252	46.7
	1–23 hr	203	37.7
	After 23 hr	85	15.6
Introduction of complementary food	Started	308	57.0
	Not started	232	43.0
Age of introduction of complementary food	0–3 months	131	24.3
	4–5 months	190	35.2
	6 months	206	38.1
	Above 6 months	13	2.4
Breastfeeding status	Still breastfeeding	485	89.8
	Stopped breastfeeding	55	10.2
Duration of breastfeeding	Before 6 months	38	7.0
	Between 6–12 months	176	32.6
	Between 13–24 months	309	57.2
	24 months and above	17	3.1

Among the mothers who introduced complementary feeding before 6 months of age, 6% did that because of the misconception that their babies were old enough to receive complementary feeding. This indicates poor maternal knowledge as these participants did not know the WHO recommendation that complementary feeding should begin only when the baby is 6 months old. The lack of compliance to the 6 months recommendations (59%) may be due to the lack of understanding of the consequences of this practice on infants' health and survival. Comparable data were reported in Anambra State, Nigeria (60%), where water and complementary foods were introduced prior to 6 months of age (Ukegbu, Ebenebe, Ukegbu, & Onyeonoro, 2011). However, these values were lower than the 80% noted for the whole country (Demographic and Health Survey and Multiple Indicators Cluster Survey DHS‐MICS, 2011). Conversely, Kakute et al. (2005) reported that all mothers in a rural area of the NWR of Cameroon introduced water and complementary foods to their children before they were 6 months old. Late introduction of complementary foods (2.4%) was higher than the 1.4% for Cameroon (C.O.L., Ministry Public Health, FECABPA., Cameroon Link and IBFAN, 2013).

#### 3.2.4 Duration of breastfeedingk

Most infants (90%) were still BF at the time the research was conducted and only 10% had stopped. As shown in table 2, most of the respondents (57%) either stopped or intended to stop BF between 13–23 months, 33% between 6–12 months, and 7% when the baby is 6 months. BF for two years and beyond is rather uncommon in this division as only 3% of mothers intended to BF their babies up to two years or beyond. This early cessation of BF deprives the children of the nutrients in breast milk and exposes the children to malnutrition (WHO, 2017). The discontinuation of BF in Momo Division increased gradually as the child grows older. Concordantly, in Bangang rural community, 2.7% of mothers continued BF their children up to 2 years or beyond (Mananga et al., 2014). This finding is not in accordance with the results reported by Karthigesu, Sandrasegarampillai, and Vasanthy Arasaratnam (2017) in Jaffna District, Sri Lanka, where 56.4% of children were breastfed for more than 2 years. A higher percentage (97.8%) of BF duration up to two years and above was reported in the southwest region of Bangladesh (Islam et al., 2013). These differences might have been caused by the mother's knowledge on recommended duration of BF which was revealed to positively affect the duration of BF (Nwachan & Ejoh, 2019; Pascale, Laure, and Enyong (2007).

#### 3.2.5 Exclusive breastfeeding

**FIGURE 2:**
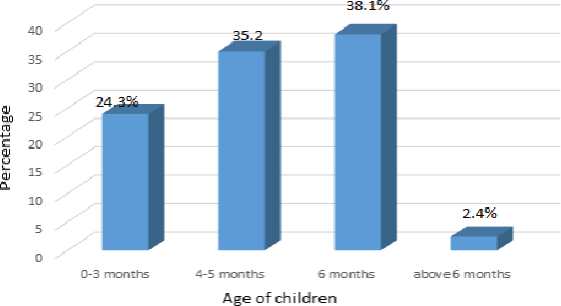
Duration of exclusive breastfeeding

As shown in Figure 2, only 38% (206) infants were exclusively breastfed until the completion of 6 months as per the WHO and UNICEF recommendations signifying a gap between the desired and the actual practice of EBF. The least percentage (2.4%) continued EBF beyond 6 months. Meanwhile, 62% of infants in Momo were deprived of the benefits of EBF as they were given complementary food or water contrary to the recommendations of WHO and UNICEF. Some of the children (24.3%) were exclusively breastfed within the periods between 0 and 3 months, while 35% of the children were exclusively breastfed within the periods between 4 and 5 months.

However, this EBF rate of 38% noticed in Momo Division is lower than the rate (52.9%) reported by Fombong et al. in 2016 for the NWR of Cameroon. This conforms to previous studies in Cameroon which show that the rate of EBF keeps on declining as years go by (Institut National de la Statistique ‐ INS/Cameroun and ORC Macro, 2005; Demographic and Health Survey and Multiple Indicators Cluster Survey DHS‐MICS, 2011; Fombong et al., 2016). Some of the reasons for this decline are because more women are getting involved in school and workforce and such mothers face difficulties in integrating EBF into their work/school schedules (Ngongalah, Rawlings, Emerson, Titilope, & Sharon, 2018).

The rate of EBF for up to 6 months noticed in this study (38%) is greater than the prevalence of 20% observed by Chiabi et al. (2011) in the West Region of Cameroon, and the national prevalence of 20% observed for Cameroon (Demographic and Health Survey and Multiple Indicators Cluster Survey DHS‐MICS, 2011) and the 23.5% shown by Chiabi et al. (2014) for women attending the vaccination and pediatric out‐patient clinics at the Yaounde Gynaeco‐Obstetric and pediatric hospital Cameroon. This low rate of exclusivity of BF noticed in this part of Africa is not surprising as it is in compliance with previous studies which revealed low rates of EBF (39%) in Africa (Cai et al., 2012), and this accounts partly for the high rate of infectious diseases such as diarrhea and pneumonia among under five children in this continent (Victora et al., 2016). Higher rates of EBF (63.9%) have been observed in Jaffna District, Sri Lanka (Karthigesu et al., 2017).

##### Reasons for not practicing EBF

**FIGURE 3:**
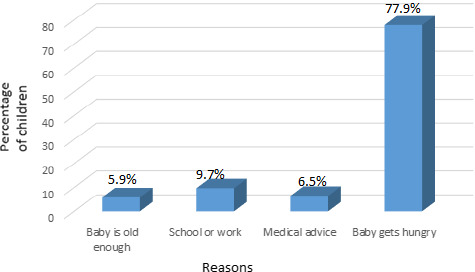
Reasons for not practicing EBF

Mothers who failed to breastfeed exclusively until 6 months gave as major reasons (Figure 3): the baby gets hungry even after BF (77.9%), medical advice (6.5%), and the mothers had to resume work or school (9.7%). In addition to these, some of the participants also had multiple misconceptions relating to duration of EBF that imply more barriers to attaining the WHO's recommendations. Such misconceptions include the beliefs that a baby below six months was old enough to eat complementary food, babies continued to be hungry after BF, babies below 6 months need water to quench their thirst and that formula‐fed infants are healthier than breastfed babies.

Other studies have also asserted these impediments to EBF (Agunbiade & Ogunleye, 2012; Cherop, Keverenge‐Ettyang, & Mbagaya, 2009; Chiabi et al., 2014; Issaka et al., 2014; Wanjohi et al., 2017).

On the other hand, mothers in a rural area of Cameroon were hindered from practicing EBF by factors such as pressure by village elders and their family since it is a traditional practice, the belief that breast milk is an incomplete food that does not increase the infant's weight and the belief that all family members should benefit from food grown in the family (Lem, 2015), while in rural Ghana the reasons were mainly family knowledge of EBF, family beliefs, and practices as major impediments to EBF (Iddrisu, 2013).

#### 3.2.6 Frequency of breastfeeding

Most mothers (79%) practiced the recommended BF frequency as they breastfed their babies more than eight times a day; meanwhile, the minority (21%) did it less than eight times a day as shown on Figure 2, 3, 4 below.

Similarly, 80% of mothers in Nigeria breastfed their children as many times as the children wanted the breast milk (Chiejina, Anieche, & Odira, 2017).

**FIGURE 4:**
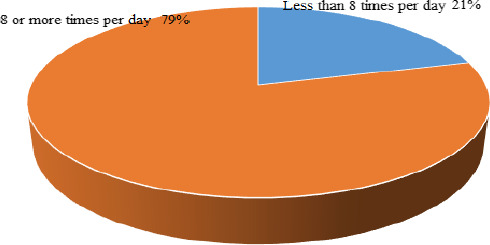
Frequency of BF

### 3.3 Maternal sources of BF information

**FIGURE 5:**
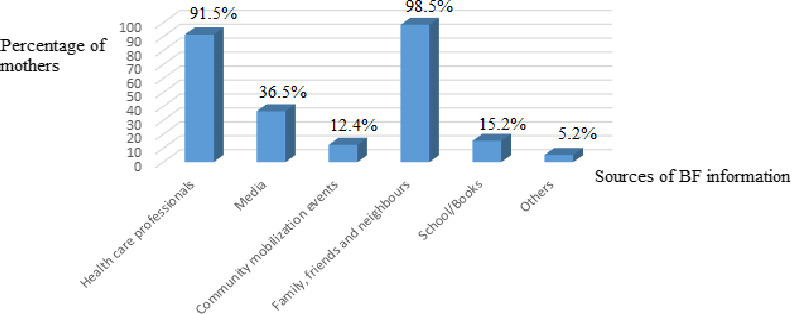
Sources of breastfeeding information for the mother

As shown in Figure 4, 5, most of the women (94.6%) received antenatal care and BF information and counseling. Only a few (5.4%) women had never received any antenatal care or information on BF. Over half of the respondents (53.1%) received BF counseling before delivery. Maternal sources of BF information are interventions by the government such as health professionals, media, school, community mobilization events (community‐based interventions to promote optimal BF) other sources like family members, neighbors, and friends. The major sources of information in this community were family members, neighbors and friends, and health facility.

The major sources of BF information in Momo Division of Cameroon, Jimma zone in Ethiopia, Kenya, Asella Town in southeast Ethiopia and Emirati are all alike and include family, friends, health professionals, radio and TV stations, (Birhanu et al., 2013; Nduati, 2012; Sasie, Oljira, & Demena, 2017; Radwan & Sapsford, 2016 respectively). The most common source of information noticed in this study which is the family, friends, and neighbors was also observed in Emirati and Asella Town, southeast Ethiopia (Sasie et al., 2017). Unlike in Momo where the most mothers received information on BF from their family members, friends, and neighbors, the most common source of information for mothers in the Okola Health District, Cameroon, is the healthcare provider (Ngameni et al., 2011).

### 3.4 Effects of sources of BF information on maternal decision

Figure 5, 6 presents the effects of diverse sources BF information the BF decision of the mothers. When ranked in order of influence on BF decision, the healthcare providers had the greatest influence on the BF decision (34%), followed by family, friends, neighbors (30%). The media (radio, television, and Internet) occupy the third position (24%), then school/book/newspaper (6%), and community mobilization events (4%). The BF decision of a few (1%) mothers was not influenced by any of the sources of information but they did what they taught was best for their children.

**FIGURE 6:**
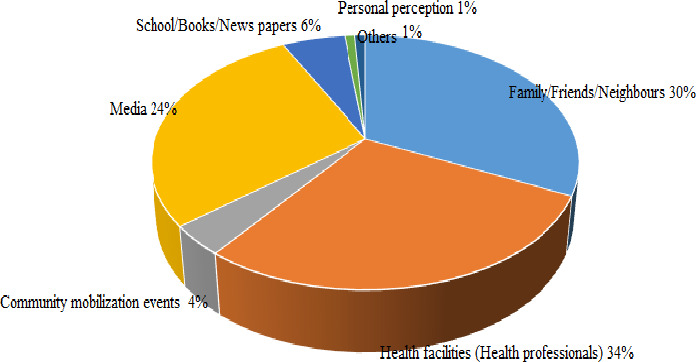
Effects of sources of information on maternal BF decision

The greatest proportion of mothers in Momo (34%) recognized interventions received from healthcare providers as the most influential intervention on their BF decisions. Similarly, most mothers in rural and urban Cameroon ranked Doctors and nurses as the most important source of advice with the greatest impact on their BF decision (Lem, 2015). Also, previous research by Castro et al. (2015) and Taveras et al. (2004) also showed that mothers often identify BF promotion interventions given by healthcare professionals as the single most important intervention that could have been offered to help them breastfeed. More so, Nguyen et al. (2018) investigated the factors that influenced the mothers' BF decision in Da Nang, Viet Nam, and found that health professionals were the most influential, followed by close family members. This is likely because health professionals are considered to be the most knowledgeable with respect to infant care practices. On the Contrary, in Emirati and Asella Town, southeast Ethiopia, the respondents recognized their own mothers, the child's father, a close relative, friend, or neighbor as the source of information with the greatest impact on maternal BF decision (Radwan & Sapsford, 2016; Sasie et al., 2017). Mothers in the Okola Health District, Cameroon, also acknowledged family members, friends, and neighbors as the source of information with the greatest impact on their BF decision (Ngameni et al., 2011). The BF decision of a few respondents (1%) like that of mothers in rural and urban Cameroon (Lem, 2015) was not influenced by any intervention but by their personal perception. Similarly, Andy (2015) showed that personally perceived seriousness, susceptibility, benefits, and barriers or cost of action are also significant determinants of maternal BF decision (Figure 6).

Some findings report differential influence on maternal decision depending on the type of educational programs used. From investigations on low‐income black Americans, it was found that while one‐to‐one educational programs were most effective in increasing initiation for women who intended to bottle feed, group programs had more impact on initiation for women who planned to breastfeed (Health Development Agency, 2003). On the contrary, it was realized that in the United States and Emirati, the family has the greatest impact on the mother's BF decision (Odom, Li, Scanlon, Perrine, & Grummer‐Strawn, 2014; Radwan & Sapsford, 2016). Also, De Oliveira, Camacho, and Tedstone (2001) indicated that the effectiveness of interventions to increase duration of any BF on maternal decision did not depend on whether they were given by health professionals or by peer counselors. He observed that peer support had a greater effect on maternal decision with regard to increasing the duration of EBF.

## 4 CONCLUSION

With regard to BF practices in Momo Division, Cameroon, compliance to most of the WHO recommendations was low. Over one‐fifth (23%) of the children were given prelacteal foods and 47% of infants put to the breast within an hour following birth. EBF (38%) for first 6 months was a rare practice as designated by early introduction of complementary foods (62%). Discontinuation of BF before the age of 24 months is a norm in this division as up to 97% of respondents ceased BF before this age.

For BF information, the mothers obtained BF information and counseling from a variety of sources including interventions by the government such as health professionals, media, school, community mobilization events, and other sources like family members, neighbors, and friends. Although the most common source of information is the family, friends, and neighbors, the most influential on maternal BF decision is the healthcare professional.

## CONFLICT OF INTEREST

The authors declare that they have no competing interests.

## ETHICAL APPROVAL

This study conforms to the Declaration of Helsinki, European Medicine Guidelines for human subjects. The study was approved by the Bamenda Regional Hospital Institutional Review Board (003/APP/RDPH/RHB/IRB, of 21/08/2017). Authorization to conduct the research was granted by the College of Technology, University of Bamenda. A verbal informed consent was obtained from all the study participants prior to their inclusion in the study.
